# DIFFERENT PATTERNS OF VACCINE HESITANCY AMONG OLDER ADULTS BY RACE/ETHNICITY, INCOME, AND EDUCATION

**DOI:** 10.1093/geroni/igad104.3283

**Published:** 2023-12-21

**Authors:** BoRin Kim, Jehoon Jeon, Chung Hyeon Jeong

**Affiliations:** University of New Hampshire, Durham, New Hampshire, United States; Eastern Connecticut State University, Willimantic, Connecticut, United States; University of New Hampshire, Durham, New Hampshire, United States

## Abstract

Despite significant efforts to increase COVID-19 vaccination rates, a substantial number of people exhibit vaccine hesitancy. Vaccine hesitancy is considered as one of the greatest threats to global health but also poses substantial risks to individuals. Moreover, vaccine hesitancy patterns may reflect and perpetuate existing health disparities. This study aims to investigate if specific patterns of vaccine hesitancy exist among older adults across racial/ethnic and socioeconomic groups. Our sample included 4,656 respondents aged 65+ who participated in the 2021 Health and Retirement Study Perspectives on the Pandemic that focuses on the first-year pandemic experiences. Latent class analysis and multinomial regression was used to identify vaccine hesitancy patterns and examine the associated factors, adjusting for age, gender, marital status, and self-rated health. Four vaccine hesitancy patterns were identified: no hesitancy (56.7%), mild hesitancy-uncertainty (19.4%), strong hesitancy-personal concerns (12.0%), and strong hesitancy-lack of trust/conspiracy (11.9%). White (RRR=1.659, p<.01), higher income (RRR=1.005, p<.05) and higher education (RRR=1.078, p<.001) were positively associated with no hesitancy. Respondents with higher education (RRR=0.573, p<.05) were less likely to be in the group of mild hesitancy with uncertainty. Hispanics (RRR=0.583, p<.05) and higher education (RRR=0.332, p<.001) were negatively associated with strong hesitancy with personal concerns. African Americans (RRR=2.426, p<.001) and those with lower education (RRR=1.324, p<.001) were more likely to be in the group of strong hesitancy with lack of trust/conspiracy. Our findings show that vaccine hesitancy is not random, which is closely related to social stratification impacting health disparities in this society.

